# *Clostridium difficile* infection in Italian urban hospitals: data from 2006 through 2011

**DOI:** 10.1186/1471-2334-13-146

**Published:** 2013-03-22

**Authors:** Stefano Di Bella, Maria Musso, Maria A Cataldo, Marcello Meledandri, Eugenio Bordi, Daniela Capozzi, Maria C Cava, Patrizia Chiaradonna, Grazia Prignano, Nicola Petrosillo

**Affiliations:** 1 National Institute for Infectious Diseases “L. Spallanzani”, Via Portuense 292, Rome, 00149, Italy; 2 S. Filippo Neri Hospital, Via Martinotti 20, Rome, 00135, Italy; 3 G.B. Grassi Hospital, Via Passeroni 28, Ostia Lido, Rome, 00122, Italy; 4 Sandro Pertini Hospital, Via Monti Tiburtini 385, Rome, 00157, Italy; 5 San Camillo-Forlanini Hospital, Circ, Gianicolense 87, Rome, 00152, Italy; 6 San Gallicano Dermatologic Institute, IRCCS IFO, Via Elio Chianesi 53, Rome, 00144, Italy

**Keywords:** *Clostridium difficile*, Epidemiology, Incidence, Italy, Hospitals, Europe

## Abstract

**Background:**

In developed countries, *Clostridium difficile* infection (CDI) represents an emerging threat in terms of morbidity and mortality rates. In our country limited CDI epidemiological data can be found.

We have conducted a 6-year retrospective study to evaluate the incidence of CDI in Italian urban hospitals.

**Methods:**

Stool samples tested for *C. difficile* toxins from January 2006 to December 2011 in 5 large hospitals in Rome, Italy, were considered in the analysis. Repeated samples taken ≤ 2 months after a positive result were excluded.

**Results:**

A total of 402 CDI episodes were identified. The incidence of CDI episodes progressively increased from 0.3 in 2006 to 2.3 per 10,000 patient-days in 2011. CDI episodes mostly occurred in patients > 60 years of age (77%). The >80 year-old age class reported the highest percentage of CDI episodes on tested samples (16%). Eighty percent (80%) of CDI episodes occurred in medical wards followed by surgery (10.2%) and intensive care units (9.8%).

**Conclusions:**

A significant increasing incidence of CDI episodes over the study period was observed during the years (p<.001), particularly in the older age groups. Medical wards experienced the highest number of CDI episodes as compared to intensive care and surgical wards. The increasing rate of CDI episodes over the last six years in our country, is alarming; urgent improvements in the surveillance systems and control programs are advisable.

## Background

*Clostridium difficile* infection (CDI) is the most frequent cause of health care-associated infectious diarrhea in industrialized countries. It predominantly affects elderly and frail hospital and nursing home patients [1,2]. Historically, the attributable mortality of CDI has been low, with death as a direct or indirect result of infection occurring in less than 2% of cases [3], however, in recent years, data from the literature show a marked increase of *C. difficile*-associated mortality and case-fatality rates [4-6].

Over the past decade, the incidence, prevalence and associated mortality of CDI have increased, particularly in North America and Europe [7-9]. In the United States (US) the incidence of adult *C. difficile*-associated diarrhea (CDAD) hospitalizations doubled from 5.5 cases per 10,000 population in 2000 to 11.2 in 2005, and the CDAD-related age-adjusted case-fatality rate rose from 1.2% in 2000 to 2.2% in 2004 [4]. In Canada, Gravel *et al*. reported a total attributable mortality rate of 5.7% among patients with nosocomial CDI [2].

Similar concerns are documented in Europe. Asensio *et al.* reported an increasing prevalence rate of CDI in Spain from 3.9 to 12.2 cases per 10,000 hospitalised patients from 1999 through 2007 [10]; Burckhardt F *et al*. reported that in Germany the incidence of CDI increased from 1.7 to 14.8 cases per 100,000 people from 2002 to 2006 [11]. In the United Kingdom (UK) a six-times increase in CDI related mortality from 1999 to 2006 was observed [6] and led to the introduction of mandatory reporting of CDI cases for surveillance purposes.

In a study conducted in 14 European countries in 2005, Barbut *et al*. reported a mean CDI incidence of 2.45 cases per 10,000 patient-days [12]; whilst a more recent European survey conducted in November 2008 collected data from 106 laboratories in 34 European countries and reported a mean CDI incidence of 4.1 per 10,000 patient-days per hospital [13]. Both studies reported the high variability in incidence of CDI among hospitals and countries.

Epidemiological data from Italy regarding CDI rates derive mainly from the European survey conducted in 2008, with only 5 Italian hospitals involved which included 57 CDI cases on 533 tested patients [13]. A small study in Bolzano, in the North of Italy, reported 13 cases of nosocomial CDI from 163 tested patients [14].

The aim of this study is to assess the trend of CDI episodes in five urban Italian hospitals over a 6-year period.

## Methods

A retrospective analysis of patients admitted to 5 urban hospitals whose laboratories participated in a network, in Rome, Italy from 2006 to 2011 was carried out. Rome municipality has around 4 million inhabitants, and the five hospitals in the study are large institutions accounting for a total of 2,677 beds (range 170–1,099), with an average of 15,481 (range 3,312-40,343) admissions per year.

All in-patients from whom stool samples were submitted to the hospital laboratory for *C. difficile* toxin detection were considered in the analysis. A CDI episode was considered as a positive *C. difficile* toxin assay in a stool sample. Repeated samples taken within two months after a positive result for any individual patient were excluded. Patients under 18 years of age were excluded from the analysis.

In the stool samples the presence of toxin A and B was tested through enzyme immunoassays for A/B toxins (Table 1). Enzyme immunoassay for *C difficile*-specific glutamate dehydrogenase and polymerase chain reaction (PCR) were not routinely performed.

**Table 1:** **Number of samples tested (total and proportion positive), and rate of *Clostridium difficile *infection (CDI) episodes per 10,000 patient-days by hospital**

**Hospital**	**CDI episodes**	**Samples tested for *C. difficile *toxins**	**CDI episodes per 10,000 patient-days**
1^*^	70	433	1.44
**2**^**‡**^	69	411	1.01
**3**^**†**^	77	813	0.39
4^*^	138	1924	1.67
**5**^**†**^	48	1370	1.62
**Overall**	**402**	**4951**	**0.87**

^*^ Meridian Immunocard (rapid EIA; Meridian Bioscence INC).

^‡^ Remel Xpect (rapid chromatographic immunoassay; Remel, Lenexa, KS, USA).

^†^ TechLab Tox A/B Quik Chek (rapid antigen capture; TechLab).

The data included patient demographics, ward and specialty at the time of sample collection, date of sample collection, type of test performed, and results.

### Statistical analysis

CDI rates are expressed as the number of patients with positive *C. difficile* toxin assay per 10,000 patient-days. A linear trend test was performed over time using the Cochran-Armitage test for trend. P values less than .05 were considered statistically significant. The analysis was done with SAS, version 9.1 (SAS Institute).

The study involved the analysis of existing clinical and laboratory data that were anonymised before being included in the study database. Thus, according to local and national regulations, this analysis was exempt from review by the Ethics Committee.

## Results

**Figure 1:**
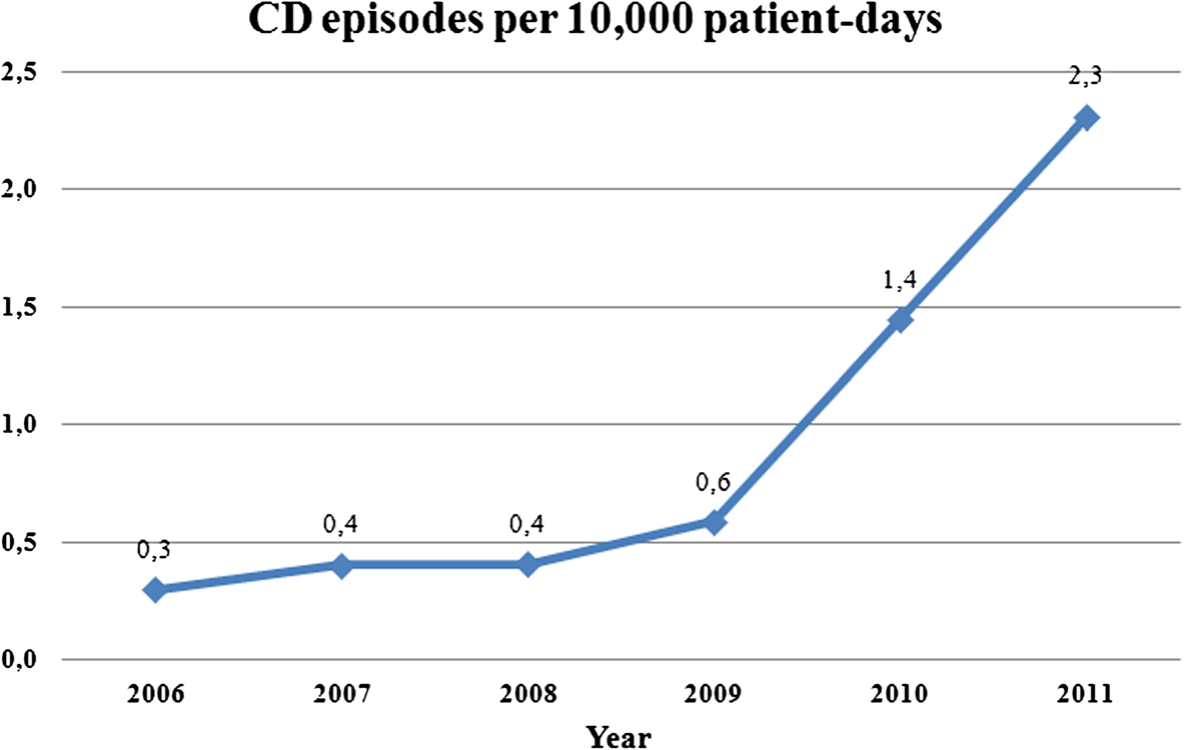
**Distribution of Clostridium difficile infection (CDI) episode incidence per 10,000 patient-days from 2006 to 2011.** Six hospitals in Rome, Italy.

**Figure 2:**
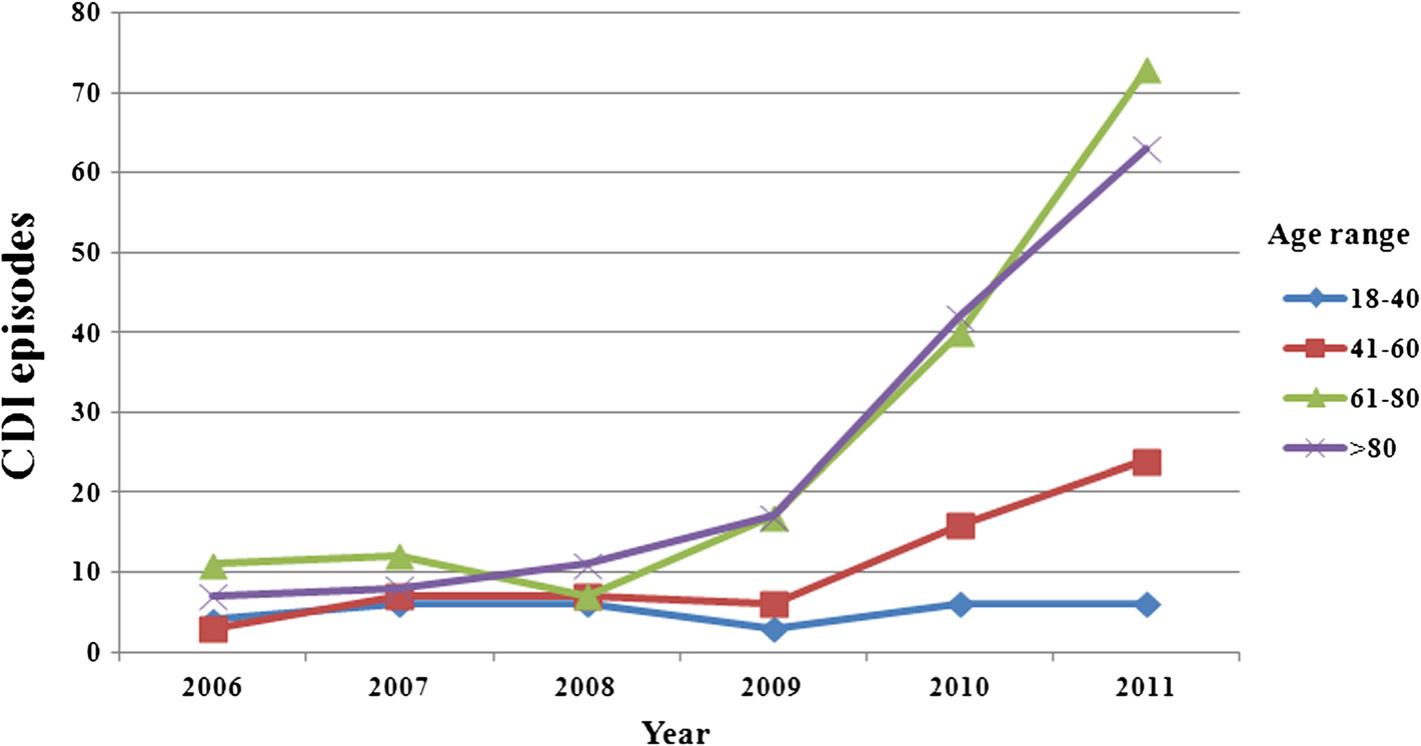
Clostridium difficile infection (CDI) episodes by year and age group.

From January 2006 to December 2011, 4951 stool samples were tested for *C. difficile* toxins in five laboratories of the hospitals participating in the study. A total of 402 CDI episodes were identified, accounting for 25, 33, 31, 43, 104, 166 in the years 2006, 2007, 2008, 2009, 2010, 2011 respectively. The incidence of CDI episodes per 10,000 patient-days significantly increased year by year, reaching the highest rate in the year 2011, as shown in Figure 1. This trend was attributable to increased number of episodes in elderly patients (Figure 2).

The overall incidence by hospital per 10,000 patient-days ranged from 0.4 to 1.7 in the study period, as shown in Table 1. However, in 2011, there was the highest rate of CDI episodes per 10,000 patient-days, i.e. 4.0, in one of the hospitals participating in the study.

**Figure 3:**
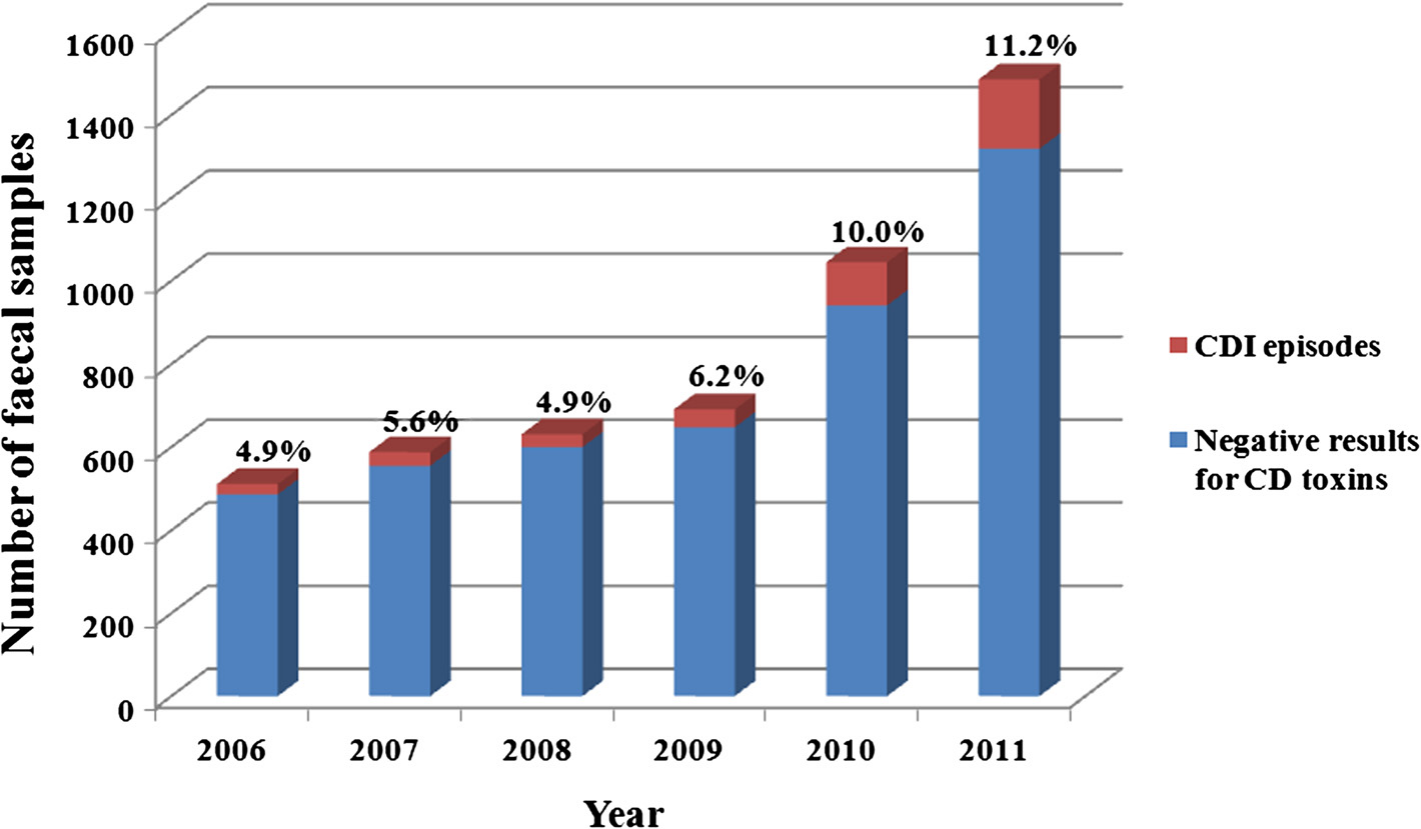
Clostridium difficile infection (CDI) proportion by stool samples assayed by year.

The number of samples assayed increased from 511 in 2006 to 1485 in 2011. The proportion of these samples identified as positive ranged from 4.9% in 2006 to 11.2% in 2011 (Figure 3).

**Figure 4:**
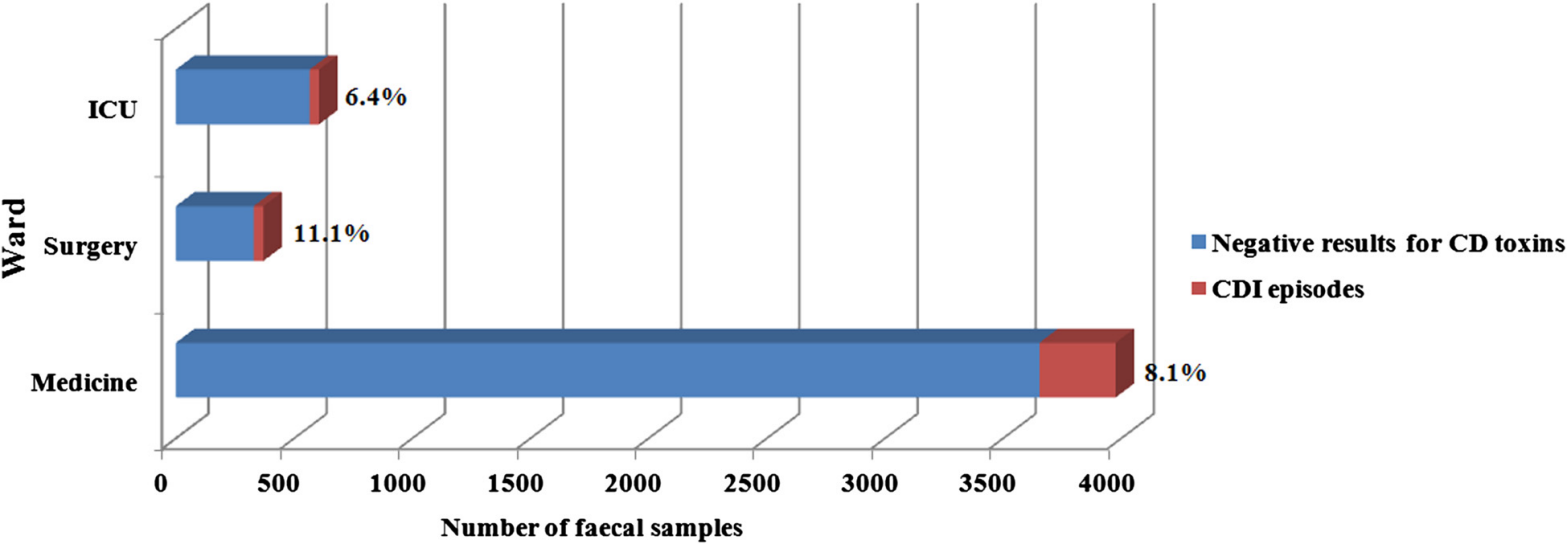
Clostridium difficile infection (CDI) proportion by stool samples assayed by ward.

Regarding specialties, the highest proportion of CDI episodes on tested samples was found in surgery (11.1%), followed by medicine (8.1%), and intensive care (6.4%). However, in terms of raw numbers of cases, medicine far outpaced others with a total of 322 CDI cases (80%) (Figure 4). Regarding the distribution of positivity by ward, 80.1% occurred in medical wards, followed by surgery (10.2%) and by intensive care units (9.7%).

The percentage of CDI episodes by stool samples increased with age from 3.4% to 16.1% in 18–40 and > 80 age groups, respectively (Table 2).

**Table 2:** Number of samples tested and CDI episodes distributed according to age groups

**Age group (years)**	**Samples tested for CD toxins**	**CDI episodes (%)**
**18-40**	920	31 (3.4)
**41-60**	1248	63 (5)
**61-80**	1866	160 (8.6)
**>80**	917	148 (16.1)
**Total**	**4951**	**402 (8.1)**

*CD* = *Clostridium difficile*.

*CDI* = *Clostridium difficile* infection.

## Discussion

Our study provides a reliable estimate of the burden of the disease in a large patient population (4951 patients), that underwent *C. difficile* toxin testing through a 6-year period in 5 urban Italian hospitals. Data from our study showed that the rate of CDI among patients undergoing *C. difficile* toxin testing is on average 0.84 per 10,000 patient-days, and that this frequency significantly increased during the years in the study, with the peak (i.e. 2.3 per 10,000 patient-days) in 2011.

Limited information on the burden of CDI in Italy is available. Italian studies mainly focus on ribotyping of collected strains or antimicrobial susceptibility testing [15]. The rate of *C. difficile* infection in Italy is still uncertain. Italian data on the incidence of CDI are derived solely from a recent European survey and a single-hospital study [13,14]. As such, the overall number of patients tested is small (<1000).

Two European studies on nosocomial CDI conducted in 2005 and 2008 reported an incidence of CDI of 2.45 per 10,000 patient-days and 4.1 per 10,000 patient-days respectively [12,13]. However both reported a wide range in incidence amongst different countries.

In the European hospital survey, 5 Italian hospitals reported a mean CDI rate per hospital of 3.6 per 10,000 patient-days [13]. This represents a four-fold higher rate compared with our average incidence rate over the period examined, but only slightly higher if compared to the 2011 rate alone (2.3 CDI episodes per 10,000 patient-days). However, the Italian population analyzed in the European survey is limited and is approximately one tenth compared to our study. Furthermore in some Italian hospitals participating in the survey, cytotoxicity test and enzyme immunoassay for *C. difficile*-specific glutamate dehydrogenase were also perfomed [13]. These test may have a higher diagnostic sensitivity. In addition, unlike in the European survey, where the CDI incidence was evaluated in a one-month period, we evaluated CDI incidence and trend over a 6-year period.

Interestingly, our trend is different from that described in the US by Lucado *et al*. [16], that reported that in the second half of the last decade the incidence rate appeared blunted. Our rising trend appears to be more similar to the first half of last decade in the Lucado study, thus hypothesizing that a delay of CDI diffusion in our region could have accounted for such a difference.

It is also of interest that in our study, there is a progressive increase in patients undergoing *C. difficile* toxin testing in the 6-year period, and a parallel progressive increase of CDI rates during the years. Increased testing could be partly related to a higher antibiotic consumption in the hospitals considered in the study, and partly to a developing CDI awareness among healthcare professionals. In recent years there has been an increasing usage of antimicrobials in the hospitals of Lazio Region, i.e. up to 34% increase from 2007 to 2009 [17]. A similar increase was found in Spain by Asensio *et al*., and was associated with the increase of CDI prevalence rates [10].

Recently a new variable has been proposed for epidemiological studies on CD: *C. difficile* testing intensity (CDTI). CDTI was defined as the total number of CD test performed, normalized to the total number of patients with at least 1 CD test recorded. The authors explored this variable with the hypothesis that part of the increase in CDI incidence is attributable to false positive related to repeated testing [18]. As a limitation of our study, data on CDTI are not available in our study population.

Our works demonstrate that the > 80 year-old age class display the highest percentage of CDI episodes, and overall the highest rates were reported in patients older than 60 years. This finding reflects similar data from previous studies conducted in the US [19] and Spain [20], where significant increasing trends between 2000 and 2003 and 1997–2005, respectively, were reported in both the older age groups with a larger increase in those aged ≥ 65 than in those aged 45–64 [19,20].

Concerning the distribution of positivity by ward, in a survey conducted in Canadian hospitals 53% of CDI occurred in medical wards (including medicine unit, long-term-care facility, oncology/hematology unit and transplant unit), 23% occurred in surgical wards and 10% in intensive care units [2], which is comparable to our findings.

Our study has some limitations. First, due to the retrospective design of the study, the clinical pictures of the patients are not available. Secondly, data on *C. difficile* testing intensity (CDTI) are not available in our study population. CDTI has been proposed as a possible factor associated with the incidence of hospital acquired CDI. CDTI, defined as the total number of CD test performed, normalized to the total number of patients with at least 1 CD test recorded, reflects the frequency of multiple test recorded for a single patient, presumably in response to an initially negative result [18]. This variable could limit the possible bias deriving from repeat testing.

Another potential limitation could be an improved sensitivity in laboratory procedures over time, leading to an increased CDI detection. However, the participating hospitals used the enzyme immunoassays for A/B toxins for the study period, and no more sensitive/specific diagnostic tool (i.e. 2-step algorithm including glutamate dehydrogenase testing and molecular biology) was adopted during the study period.

Data concerning antimicrobial consumption in the surveyed hospitals during the study period are lacking. We were unable to assess the rate of CDI episodes per patient-days by ward, due to the unavailability of the number of admissions per ward. Finally, data analised according to subspecialty are not available either.

## Conclusions

The increasing rate of CDI episodes in Italian hospitals over the last six years represents a matter of concern for our country; the emergence of hypervirulent *C. difficile* strains, already circulating in Italy [21], is a cause of further worry. An urgent need for improvements in the surveillance systems and infection control programs, including antimicrobial stewardship strategies, is advisable in our hospitals.

## Competing interests

None of the following people, DBS, MM, CMA, MM, BE, CD, CMC, CP and PG. PN received honoraria as speaker from: Pfizer, Wyeth, Sanofi Aventis, Astellas, MSD, Gilead, Novartis, GSK, Johnson & Johnson, Jansen Cilag, and as member of scientifical board from MSD, Pfizer and Carefusion.

## Authors’ contributions

DBS, MM^*^, CMA and PN participated in the design of the study; DBS and PN performed the statistical analysis. MM^#^, BE, CD, CMC, CP and PG participated in the acquisition of data. DBS, MM^*^ and PN participated in its coordination and helped to draft the manuscript. All authors read and approved the final manuscript.

## Pre-publication history

The pre-publication history for this paper can be accessed here:

http://www.biomedcentral.com/1471-2334/13/146/prepub
